# Effects of a Bovine Lactoferrin Formulation from Cow’s Milk on Menstrual Distress in Volunteers: A Randomized, Crossover Study

**DOI:** 10.3390/ijms17060845

**Published:** 2016-05-31

**Authors:** Hiroshi M. Ueno, Ran Emilie Yoshise, Tomohiro Sugino, Osami Kajimoto, Toshiya Kobayashi

**Affiliations:** 1 Milk Science Research Institute, Megmilk Snow Brand Co., Ltd., Saitama 350-1165, Japan; hiroshi-ueno@beanstalksnow.co.jp (H.M.U.); yr-emilie@meg-snow.com (R.E.Y.); 2 Soiken Inc., Osaka 560-0082, Japan; sugino@soiken.com; 3 Graduate School of Medicine, Osaka City University, Osaka 545-8585, Japan; kajimoto@med.osaka-cu.ac.jp

**Keywords:** lactoferrin, menses, distress, iron, complex

## Abstract

Dysmenorrhea is a highly prevalent complaint and highly undiagnosed gynecologic condition. Dairy products have a potential in the management of menstrual distress, and bovine lactoferrin can help the subjective dysphoria associated with dysmenorrhea. In the present study, we aimed to investigate the effects of a lactoferrin formulation isolated from cow’s milk on menstrual symptoms in volunteers. A double-blind, randomized, placebo-controlled, crossover study of the iron-lactoferrin complex (FeLf) was performed in thirty-five healthy Japanese women. Participants received the 150 mg FeLf (per day) or placebo from day ten of the luteal phase to day four of the follicular phase. The Moos Menstrual Distress Questionnaire (MDQ) was measured for menstrual distress, and heart rate variability was measured as an index of autonomic nerve balance during menses. A visual analog scale for menstrual pain, and a verbal rating scale for quality of life during the first three days of menstruation were measured. The MDQ score for the automatic nervous system subscale was lower and the parasympathetic nervous system activity was greater in FeLf than in placebo for intention-to-treat or per-protocol populations. The other variables were not different between the groups. No treatment-related side effects were observed during the study. The results indicate that FeLf can provide a beneficial effect on the psychological symptoms in women affected by menstrual distress.

## 1. Introduction

Dysmenorrhea is a highly prevalent complaint, and results in work or school absenteeism in women of reproductive age, whereas the prevalence of dysmenorrhea varies between 16% and 91%, with severe pain in 2%–29% of the women [1]. Although conventional agents, such as nonsteroidal anti-inflammatory drugs (NSAIDs), show good effectiveness, they offer inadequate treatment because 20%–25% of women experience inadequate relief of dysmenorrhea despite treatment [2]. In addition, NSAIDs are often associated with adverse effects, such as stomach upset. In Japan, about one-third of women suffer from severe dysmenorrhea [3], but half of these self-medicate, and some women prefer to use complementary/alternative medicines [4,5].

Diet is a modifiable lifestyle factor of dysmenorrhea, and there are several plausible connections between diet and dysmenorrhea [6]. Dysmenorrhea is related to a low fish intake, and high n-3 fatty acid intake and a high n-3/n-6 fatty acids ratio decrease menstrual pain, indicating that fish oil intake can lower dysmenorrhea. Dietary fiber also shows inverse correlation with menstrual pain. In an epidemiological study, dysmenorrhea and associated symptoms were found in significantly fewer adolescent college students who consumed three or four servings of dairy products per day as compared to women who consumed no dairy products [7]. Dietary calcium comes largely from dairy products, so that calcium plays a possible protective role in the management of primary dysmenorrhea. On the other hand, other dairy components have a potential to alleviate the symptoms associated to dysmenorrhea.

Lactoferrin (Lf) is an iron-binding glycoprotein belonging to the transferrin family, and is present in milk and secretory fluids [8,9]. In response to inflammatory conditions, peripheral (plasma) Lf is secreted from neutrophils, and microglia secrets Lf in the central nervous system. Bovine Lf may be administered via oral, intraperitoneal, or intrathecal routes. In rats, Lf is reported to have antinociceptive effects [10], analgesic effects [11], suppressive effect on distress [12] in dose-dependent manner. Moreover, two preliminary studies revealed that the oral administration of Lf (300 and 600 mg of Lf daily) ameliorated the subjective dysphoria associated with dysmenorrhea [13,14]. A recent trial indicates that the clinical efficacy of bovine Lf is effective for the common cold at 200 mg/day with a combination of bovine immunoglobulin from cow’s milk in healthy volunteers [15]. The effective dosage for healthy volunteers might be lower than that in the previous studies at 300 and 600 mg of Lf daily.

In the present study, we investigated the effects of an Lf preparation on mense-induced symptoms using an iron-Lf complex (FeLf) in a double-blind, randomized, placebo-controlled, crossover study. Because the formation of iron-binding complexes increases the stability of bovine Lf, FeLf is more stable than Lf to enzymatic hydrolysis and thermal- or detergent-induced denaturation [16,17,18]. Thus, we hypothesized that oral FeLf is well tolerable and provides effective relief from menstruation-associated symptoms. To test this hypothesis, we examined the effectiveness of oral FeLf (123.8 mg of Lf daily) on subjective and objective measures of menstrual distress in women during menses.

## 2. Results

### 2.1. Participant Disposition and Baseline Characteristics

**Figure 1:**
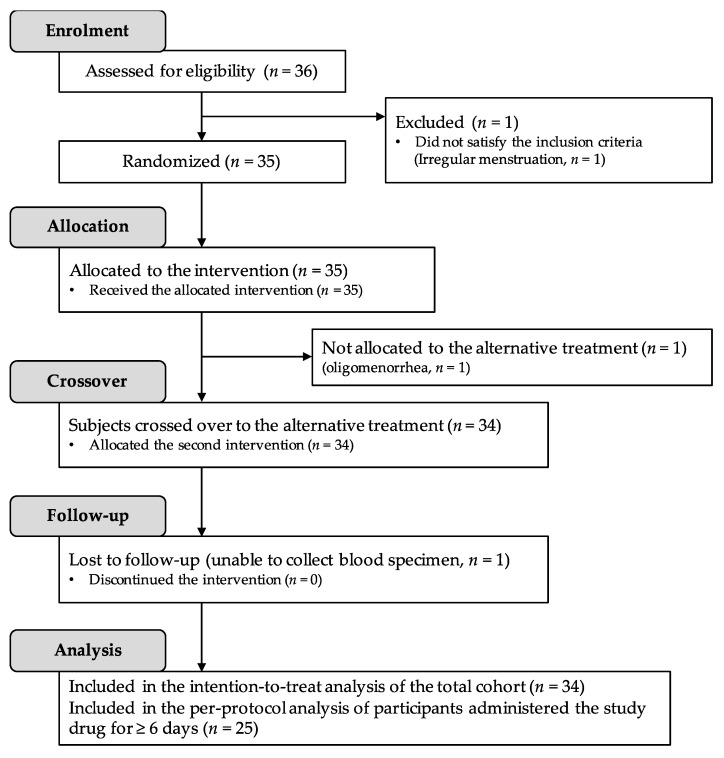
Participant disposition.

Figure 1 shows the disposition of the participants. A total of 36 healthy women were initially enrolled into the study. One woman was excluded because of irregular menstruation. Therefore, 35 were randomized to receive FeLf or placebo tablets in a double-blind manner for their initial menstrual period. After a washout period of one menstrual cycle, the participants were crossed over to the alternative intervention. One woman withdrew during the study because of oligomenorrhea, although no relationship was observed between the symptoms and her participation in the study. Thirty-four women completed both study periods; one woman did not provide a blood specimen because of a temporary mood disorder at the time of blood sampling. The intention-to-treat analysis was performed on all participants (*n* = 34) who underwent randomization and took the product (placebo and FeLf). Finally, the responses of 25 healthy volunteers were retained for the per-protocol analysis.

Table 1 shows the baseline characteristics, including age, body mass index, finger photoplethysmographic waveform variability, and iron-related laboratory variables (hemoglobin, hematocrit, serum iron, and serum ferritin), for the participating women. Twenty-five women were included in the per-protocol analyses; the other nine women were excluded because they administered the study drug for <six days per menstrual cycle because of the early onset or termination of menses. None of the participants reported any adverse effects during the interventions.

### 2.2. Subjective Symptoms

In the intention-to-treat cohort, the Moos Menstrual Distress Questionnaire (MDQ) score for the autonomic nervous system subscale improved significantly in the FeLf group compared with the placebo group (1.1 *vs.* 1.8, *p* = 0.025; Table 2a). However, there were no significant differences between the two groups in the other MDQ subscales, or the visual analogue scale (VAS) score for menstrual pain or the VRS score for quality of life (Table 2a).

In the per-protocol subgroup of women who administered the study drugs for ≥six days, the MDQ score for the autonomic nervous system subscale improved significantly in the FeLf group compared with the placebo group (1.0 *vs.* 1.9, *p* = 0.006; Table 2b). The MDQ scores for fluid accumulation and the negative feeling subscales were also lower in the FeLf group than in the placebo group, although the differences were not statistically significant (fluid accumulation: 2.4 *vs.* 3.0, *p* = 0.084; negative feelings: 1.8 *vs.* 3.2, *p* = 0.059; Table 2b). There were no significant differences in other MDQ subscale, VAS, or verbal rating scale (VRS) scores between the two groups (Table 2b).

### 2.3. Heart Rate Variability

Heart rate variability is an objective index of the autonomic nerve balance involved in the psychological response to a stressor. Heart rate variability was measured by finger photoplethysmographic waveform variability using acceleration plethysmography [19] before and after menstruation, and the results are shown in Table 2. Using this method, a reduction in HFA or an increase in LFA indicates the predominance of sympathetic activity. In the intention-to-treat analysis, H% was significantly greater in the FeLf group than in the placebo group (41.1% *vs.* 31.7%, *p* = 0.024; Table 2a). In the per-protocol analysis, H% tended to be higher and LFA/HFA tended to be lower in the FeLf group than in the placebo group (H%: 41.9% *vs.* 32.1%, *p* = 0.051; LFA/HFA: 1.076 *vs.* 2.051, *p* = 0.069; Table 2b). The other variables were not significantly different between the two groups in the intention-to-treat or the per-protocol population.

## 3. Discussion

We conducted a randomized, double-blind, placebo-controlled crossover study to evaluate the effects of FeLf in relieving subjective dysphoria in women during menses. No side effect was observed by the intervention throughout the study, suggesting that FeLf was well tolerable in women during menses. We found that oral FeLf improved the MDQ score for the autonomic nerve balance scale and heart rate variability (Table 2) compared with the placebo. These findings are the first to show that FeLf improves subjective and objective symptoms of menses. Our previous study showed that FeLf was appeared to palliate the distress against the calculation work; a single oral intake of FeLf (833 mg) alleviated the changes of subjective score of fatigue, salivary IgA and CgA, and brain wave during the calculation work [20]. Taken together, the oral administration of FeLf appeared to lessen the distress against tentative stressor within a few days. In psychiatry, psychological responses are often assessed using objective methods. Several studies have reported that a person with depressive symptoms often shows a predominance of sympathetic activity corresponding to a reduction in the H component or an increase in the L/H ratio on heart rate variability analysis [21,22]. The heart rate variability analyses in this study indicated that FeLf induced a predominance of the parasympathetic nervous system compared with placebo. In the menstrual cycles of healthy women, the sympathetic nervous system is dominant in the luteal phase and the parasympathetic nervous system is dominant in the follicular phase. Thus, oral FeLf seemed to regulate autonomic function during menstruation in these women during menses. The psychological responses measured with acceleration plethysmography were significantly different between the two interventions in the intention-to-treat cohort, whereas the effects of FeLf on the subjective symptoms in terms of the MDQ subscores tended to be greater in the per-protocol population. These findings suggest that the effects of FeLf were greatest in women administered it for ≥six days during their menstrual period. Since the duration of menses generally lasts within seven days, continual intake at least six days would be appropriate to achieve the effect of FeLf. An epidemiological survey revealed that only 20% of Japanese women visit a gynecologist, even though 74% suffer from menstrual dysphoria [23], and that most of over-the-counter drugs used during menstruation are analgesics. These findings imply that menstrual symptoms other than pain are unlikely to improve during palliative treatment. The safety of Lf is widely accepted and it is present in many conventional foods, including dairy products, dietary supplements, and infant formulae [24,25]. Because Lf is found in milk and dairy products, habitual dietary supplementation with Lf is a promising way of dietary management with dairy products to palliate menstrual distress in women. With the modification of a brain-targeting ligand, a brain delivery vehicle can be available as well to improve the effect of lactoferrin as neutraceuticals [26].

A limitation of this study was the criteria for volunteers. We defined inclusion criteria as the presence of pain during menstruation because Japanese women tend to cope with the symptoms of dysmenorrhea without medication and prefer to self-administer analgesics to relief the symptoms associated with menses. FeLf was ineffective in terms of relieving menstrual pain according to the VAS scores (Table 2). Considering the VAS score was corresponding to severe pain above 55 mm [27], the large variation in the VAS measurements might be difficult to determine the efficacy of FeLf on the menstrual pain. Dysmenorrhea improves with age, parity, and the use of oral contraceptives and is positively associated with stress and a family history of dysmenorrhea [1]. Therefore, the intensity of menstrual pain may be more strongly affected by factors other than FeLf. Meanwhile, the use of NSAIDs was a possible confounding factor in this study; however, NSAIDs was popular medication in daily medical practice and self-mediation. Thus, we allowed the volunteers to use NSAIDs as a rescue treatment and measured its effect by the VRS scoring in this study. We found that the VRS score that could reflect the use and efficacy of painkillers in our preliminary study [14]. In the present study, the VRS score was not significantly different between the two groups in the intention-to-treat and per-protocol analyses, while four participants used painkillers in the intention-to-treat analysis including three participants in the per-protocol analysis (Table 2). Accordingly, painkillers would not have a significant effect in this study. Assuming that the central problem with somatization is pain, the dose-dependency should be further evaluated in order to compare the outcomes of preliminary studies. In addition, because the severity of menstrual pain varies between individuals, it should be considered part of the eligibility criteria for studies like ours. Some clinical trials have examined the safety and efficacy of Lf for the treatment of iron disorders in women of reproductive age. Notably, Lf is more effective and safer than ferrous sulfate for treating iron deficiency and iron deficiency anemia [28]. In the present study, the baseline values and changes in iron-related variables were not significantly different between the FeLf and placebo groups, and indicated that iron status was normal in the participants. Although iron supplementation improves fatigue among women with nonanemic iron depletion and low serum ferritin levels [29,30], the effectiveness of iron intake may be affected by the baseline characteristics of the subjects, as well as the dose and duration of treatment. In women with normal iron and ferritin concentrations, the short-term administration of supplemental iron hardly affects their hematological parameters, such as the red blood cell count, hemoglobin, hematocrit, and serum iron. In addition, Japanese women of reproductive age take iron approximately 6 mg/day according to the governmental survey, and the Japanese dietary guideline recommends taking iron at 10.5–11.0 mg for those populations [31,32]. Therefore, 150 mg of FeLf containing 7.5 mg iron per day was a possible supplementation of iron for the healthy populations.

## 4. Materials and Methods

### 4.1. Participants

This randomized, double-blind, placebo-controlled crossover study was conducted at Soiken Inc. (Osaka, Japan). The study was approved by the local institutional review board of Fukuda Clinic (Osaka, Japan), and was registered with the Japanese University Hospital Medical Information Network clinical trials registry (UMIN000013220). Each participant provided her written informed consent. Women living in the Kansai region of Japan were enrolled between January and June 2012.

Healthy women aged 20–49 years were eligible if they had regular menstrual cycles (28 ± 2 days), had no allergies to dairy products or Japanese cedar, and had not received medical (e.g., prescription drugs) or surgical treatment for up to eight weeks before enrollment. Only women who were aware of subjective dysphoria at menses, as defined by the presence of pain during the menstrual phase, were enrolled. Any other criteria, such as obstetrical, medical and surgical histories were not included in this study.

### 4.2. Procedure

The participants were randomly assigned to receive either FeLf or placebo during the initial menstruating period and were then crossed over to the alternative treatment in the subsequent menstruating period. In the FeLf arm, participants took two 330 mg tablets (150 mg FeLf; 6.7 mg of iron and 123.8 mg of Lf from bovine milk per day) every day from day ten of the luteal phase to day four of the follicular phase. The menstrual cycles were determined based on their basal body temperature. In this study, the menses defined as any spotting or bleeding from uterus. In the placebo arm, the participants took two tablets with the same composition as the FeLf tablet, except FeLf was replaced with cellulose, maltose, and colorant. FeLf (Lf: 82.5% *w*/*w*; iron: 4.5% *w*/*w*) was obtained from Megmilk Snow Brand (Tokyo, Japan), and was prepared by blending Lf with iron (III) chloride in the presence of sodium hydrogen carbonate, followed by desalting and freeze drying, as previously described [15]. The FeLf and placebo tablets were prepared by API Co., Ltd. (Gifu, Japan) in ten grams (approximately 30 tablets) packs and were identical in appearance. The compositions of the test tablets are shown in Table 3. The use of NSAIDs was allowed during the study, but participants using other medical treatments for menstrual pain or physical complaints were withdrawn from the study. Randomization was conducted by an independent statistician (Statcom Co., Ltd., Tokyo, Japan), and was stratified by age and body mass index to ensure that these variables were not significantly different between the two groups. The independent statistician prepared a computer-generated randomization schedule according to a descending order of the randomly generated number corresponding to each participant. The trial was performed in a double-blind manner in which the allocated drug and measured data were concealed from all study investigators and participants throughout the study.

The study outcomes were subjective and objective measures of menstruation, which were assessed in each menstrual cycle. The intensities of psychological symptoms were assessed using the MDQ [33]; menstrual pain was assessed using a VAS [34]; and quality of life was assessed using the VRS [13,14]. In the MDQ, the participants were asked to rate their experience for 47 symptoms using a six points scale ranging from no experience of the symptom to acute or partially disabling experience of the symptom. The VAS was presented as a ten centimeters line, anchored by the verbal descriptors “no pain” and “worst imaginable pain” [35]. The VRS comprises a list of questionnaires regarding daily life, which included the difficulty in performing schoolwork, business work, and housework, dysphoria except for pain, and the frequency of using analgesic drugs. Each item was scored from zero (nothing) to three (very often), except for dysphoria (zero or one; absence or presence). The MDQ, VAS, and VRS were to be recorded at bedtime during the menstrual period, from day ten of the luteal phase to day four of the follicular phase. The participants also recorded their body temperatures, food intake, and the use of NSAIDs. Blood samples were drawn for hematological tests (red blood cell count, hemoglobin, and hematocrit) and the measurement of serum iron and ferritin concentrations.

The heart rate variability indices of low-frequency area (LFA), high-frequency area (HFA), ratio of the power values for LFA/HFA, and the power values of the low-frequency (L%) or high-frequency (H%) areas relative to the total power area (TP) were measured by finger photoplethysmographic waveform variability using acceleration plethysmography (Artett C; U-medica Inc., Osaka, Japan), as previously described [19,36]. The machine automatically calculates the coefficient of variation for the a–a intervals obtained from the acceleration plethysmograms to estimate autonomic nerve activity. The maximal entropy method was used to analyze the frequency spectrum.

### 4.3. Sample Size, Data Analyses, and Statistical Tests

To detect a difference in the menstrual pain score of 15 points between the two groups, and assuming a mean pain score of 42 points and a standard deviation of 18 points, according to our preliminary study [14], the required sample size was estimated to be 24 participants per group at α = 0.05 and β = 0.2 (power: 80%). Because we conducted a placebo-controlled crossover study, a total of 24 participants were required. We recruited 36 women; 35 women started the intervention and 34 women completed the study. The intention-to-treat analysis was performed on the completed participants. We conducted a per-protocol analysis of women who were administered FeLf for ≥six days in accordance with menses, because the administration period of FeLf affected the subjective measures according to the subgroup analysis in our preliminary study [14]. The mean changes in each variable from baseline to the final evaluation within each treatment period were compared between the two interventions using Student’s *t* test or the Wilcoxon two-sample test. *p* values of < 0.05 were considered statistically significant and the actual p values are stated for all variables with *p* values of < 0.10. IBM SPSS statistics version 11.5 (IBM Corp., Armonk, NY, USA) was used to analyze the data.

## 5. Conclusions

The consumption of FeLf was found to result in the significant improvement of stress-induced psychological symptoms (a MDQ subscore for autonomic balance, heart rate variability). On the other hand, iron supplementation did not significantly affect iron status in healthy women with mense. The results indicate that FeLf can provide a beneficial effect on psychological symptoms in women affected by menstrual distress.

## Figures and Tables

**Table 1:** Baseline characteristics of the participants (*n* = 35) ^1^.

Variable	Value (Range)
Age (year)	36.9 ± 3.9 (27–45)
Body weight (kg)	57.3 ± 9.7 (44.8–93.7)
Body mass index (kg·m^−2^)	22.3 ± 3.6 (18.4–35.7)
Serum iron (μg·dL^−1^)	94.9 ± 54.1 (20–219)
Serum ferritin (ng·mL^−1^)	28.5 ± 29.0 (5.0–115.1)
Red blood cell count (×10^4^ μL^−1^)	444 ± 31 (369–494)
Hemoglobin (g·dL^−1^)	12.8 ± 1.2 (9.8–15.2)
Hematocrit (%)	38.2 ± 2.8 (30.1–44.4)

^1^ Data are presented as means ± standard deviations.

**(a):** Intention-to-Treat Analysis.

Measurements		FeLf (*n =* 34)	Placebo (*n =* 34)
MDQ subscale	Pain	6.2 ± 4.0	6.5 ± 4.3
	Concentration	2.6 ± 4.7	3.3 ±5.0
	Autonomic nervous system	1.1 ± 1.7 *	1.8 ± 2.2
	Behavior change	4.1 ± 4.6	4.0 ± 4.6
	Fluid accumulation	2.9 ± 2.6	3.2 ± 3.1
	Negative feeling	1.9 ± 3.1	2.5 ± 4.1
	Hyperthymia	1.6 ± 3.4	1.5 ± 3.2
	Control	0.7 ± 1.5	1.0 ± 1.8
Finger photoplethysmographic waveform variability	LFA ^2^	709.1 ± 669.2	836.1 ± 772.7
	L% ^3^	32.5 ± 15.5	33.7 ± 14.7
	HFA ^4^	941.6 ± 918.4	695.7 ± 598.8
	H% ^5^	41.1 ± 18.8 *	31.7 ± 15.0
	TP ^6^	2175.2 ± 1587.6	2449.2 ± 2530.4
	LFA/HFA ^7^	1.3 ± 1.6	1.9 ± 2.7
Menstrual pain		53.5 ± 32.7	50.6 ± 35.5
Quality of life		2.1 ± 2.0	2.0 ± 1.9

**(b):** Per-Protocol Analysis.

Measurements		FeLf (*n =* 25)	Placebo (*n =* 25)
MDQ subscale	Pain	6.1 ± 4.3	6.8 ± 4.7
	Concentration	2.5 ± 4.7	3.5 ± 5.7
	Autonomic nervous system	1.0 ± 1.7 **	1.9 ± 2.4
	Behavior change	4.0 ± 4.6	4.8 ± 5.0
	Fluid accumulation	2.4 ± 2.5 ^†^	3.0 ± 3.1
	Negative feeling	1.8 ± 3.4 ^†^	3.2 ± 4.6
	Hyperthymia	2.2 ± 3.9	1.9 ± 3.6
	Control	0.8 ± 1.7	1.1 ± 2.0
Finger photoplethysmographic waveform variability	LFA	627.4 ± 623.2	760.5 ± 829.5
	L%	30.3 ± 13.4	32.3 ± 15.3
	HFA	940.5 ± 991.3	610.8 ± 552.0
	H%	41.9 ± 18.9 ^†^	32.1 ± 16.9
	TP	2118.2 ± 1750.9	2023.1 ± 1291.3
	LFA/HFA	1.1 ± 1.0 ^†^	2.1 ± 3.1
Menstrual pain		53.9 ± 33.7	54.0 ± 36.2
Quality of life		2.0 ± 2.0	2.2 ± 1.7

^1^ Data are presented as means ± standard deviations (^†^ *p* < 0.10, * *p* < 0.05, ** *p* < 0.01); ^2^ LFA: low-frequency area; ^3^ L% = power value for the low-frequency area relative to the total power area; ^4^ HFA = high-frequency area; ^5^ H% = power value for the high-frequency area relative to the total power area; ^6^ TP = total power; ^7^ LFA/HFA = ratio of L to H (L/H).

**Table 3:** Compositions of the iron-lactoferrin complex (FeLf) and placebo tablets ^1^.

Nutrients	FeLf	Placebo
Iron (mg)	6.7	0.0
Lactoferrin (mg)	123.8	0.0
Energy (kcal)	2.5	2.6
Protein (g)	0.1	0.0
Fat (g)	0.0	0.0
Carbohydrate (g)	0.5	0.6
Sodium (mg)	5.6	2.5

^1^ Values are shown for the daily dose of FeLf and placebo.

## Abbreviations

Lf	lactoferrin
FeLf	iron-lactoferrin complex
MDQ	Moos menstrual distress questionnaire
NSAID	nonsteroidal anti-inflammatory drug
VAS	visual analogue scale
VRS	verbal rating scale
HFA	heart rate variability index of high-frequency area
LFA	heart rate variability index of low-frequency area
H%	power value of the high-frequency relative to the total power area
L%	power value of the low-frequency relative to the total power area
